# ORIF versus arthroplasty for open proximal humerus fractures: Nationwide Inpatient Sample data between 1998 and 2013

**DOI:** 10.1186/s10195-018-0503-1

**Published:** 2018-08-22

**Authors:** Anant Dixit, Frank S. Cautela, Colin S. Cooper, George A. Beyer, James C. Messina, Jeffrey E. Mait, Neil V. Shah, Bassel G. Diebo, Carl B. Paulino, William P. Urban

**Affiliations:** Department of Orthopaedic Surgery and Rehabilitation Medicine, State University of New York, Downstate Medical Center, 450 Clarkson Avenue, Box 30, Brooklyn, NY 11203 USA

**Keywords:** ORIF, Arthroplasty, Shoulder, Proximal humerus, Open fracture, Nationwide Inpatient Sample (NIS)

## Abstract

**Background:**

Limited data exists in analyzing open reduction and internal fixation (ORIF) and arthroplasty in the management of open proximal humerus fractures. We analyzed differences in hospital course between these procedures, patient demographics, complication rate, length of stay, hospital charges, and mortality rate.

**Materials and methods:**

This is a retrospective review of the Nationwide Inpatient Sample database. ICD-9 codes identified patients hospitalized for open proximal humerus fractures from 1998 to 2013 who underwent ORIF or shoulder arthroplasty (hemi-, total, or reverse). Demographics and in-hospital complications were compared. Logistic regression controlling for age, gender, and Deyo index tested the impact of ORIF vs ARTH on any complications.

**Results:**

Seven hundred thirty patients were included (ORIF, *n* = 662 vs ARTH, *n* = 68). ORIF patients were younger (*p* < 0.001), more likely to be males (*p* < 0.001), and had a lower Deyo score (*p* = 0.012). Both groups had comparable complication rates (21.4% vs 18.0%, *p* = 0.535), lengths of stay (7.86 days vs 7.44 days, *p* = 0.833), hospital charges ($76,998 vs $64,133, *p* = 0.360), and mortality rates (0.2% vs 0%, *p* = 0.761). Type of surgery was not a predictor of any complications (OR = 0.67 [95% CI 0.33–1.35], *p* = 0.266), extended length of stay (OR = 1.01 [95% CI 0.58–1.78], *p* = 0.967), or high hospital charges (OR = 1.39 [95% CI 0.68–2.86], *p* = 0.366).

**Conclusion:**

We revealed no differences in hospital course between ORIF and arthroplasty for management of open proximal humerus fractures. Although differences in demographics existed, no differences in complication rates, length of stay, hospital charges and mortality rates were noted. Future studies can evaluate the long-term outcomes of these procedures.

**Level of evidence:**

Level III.

## Introduction

Proximal humerus fractures are the third most common fracture in adults, representing almost 6% of all adult fractures and half of fractures of the humerus [1–3]. They are typically associated with minimal trauma in the elderly, particularly in osteoporotic individuals, and with high-energy trauma in younger patients. Most of these fractures can be treated conservatively with satisfactory results [4]. More complex fractures may require surgical intervention, such as with open reduction and internal fixation (ORIF) or arthroplasty (either total, reverse total, or hemiarthroplasty). Currently, there is conflicting data in the literature with regards to which surgical technique is most effective at treating fractures of the proximal humerus. Even though it appears as if ORIF is the technique preferred for younger patients, there is no consensus on which treatment option is most preferred or effective for all patients [5]. While it has been shown to demonstrate better functional outcomes and restoration of anatomy, ORIF can also be associated with higher reoperation rates [6, 7].

Open proximal humerus fractures in particular are a relatively rare but serious injury. One study found that out of 1582 proximal humerus fractures over a 2-year period, only 3 (0.2%) were open [8]. Open humeral fractures in general tend to be associated with high energy trauma, such as motor vehicle accidents [8, 9]. These injuries can be associated with more serious sequelae, such as injury to the axillary artery [10]. Due to the fact that they are relatively uncommon, there is a paucity of literature analyzing specifically open proximal humerus fractures and management.

Due to the conflicting nature of the literature, lack of data, and the importance of the topic, we utilized the Nationwide Inpatient Sample (NIS) database to analyze the differences in the hospital course of patients who were treated with ORIF vs arthroplasty for open fractures of the proximal humerus. Specifically, we sought to do this in terms of demographics, complication rate, length of stay, hospital charges, and mortality rate.

## Materials and methods

### Data source

All data was used from a subset generated from the Nationwide Inpatient Sample (NIS) spanning the years 1998–2013. NIS is supported by the Health Care Cost and Utilization Project (HCUP) and sponsored through both federal, state, and industry partnerships. The NIS database provides the most robust, publicly available data for inpatient care across all-payers in the United States. In its entirety, more than seven million individual hospital visits per year were recorded within the data set. Our institutional review board approved this study as exempt due to the de-identified nature of the data. Inclusion criteria were patients admitted with ICD-9 codes of primary diagnoses of open proximal humerus fractures (812.10, 812.11, 812.12, 812.13, 812.19). Patients with multi-trauma or pathologic fractures were excluded.

### Data collection

The demographics studied include age, gender, race, Deyo index, and type of insurance as reported in the NIS dataset. Using ICD-9 procedure codes, patients that underwent either ORIF (79.31) or arthroplasty (reverse 81.88, total 81.80, partial 81.81) following open proximal humerus fractures were grouped into the corresponding subset. In-hospital complications were grouped based on related sets of diagnoses as follows: infection, post-hemorrhagic anemia, cardiac complications, respiratory complications, device-related complications, post-operative shock, hematoma formation, pulmonary insufficiency, deep vein thrombosis (DVT), pulmonary embolism (PE), and any complication. Any complication was defined as the occurrence of at least one complication. Length of hospital stay, total cost of stay, and mortality were also reported.

### Study design and statistical analysis

Descriptive analysis was used to characterize the open proximal humerus fracture population. Patients were stratified based on the surgical repair; ORIF patients comprised the first group, while total, hemi, and reverse arthroplasty patients were combined into a second group. Independent sample *t*-tests were employed to evaluate variation of continuous variables such as age, Deyo index, and total hospital cost between the two groups. Accordingly, chi-square analysis was used to evaluate the potential association between gender, race, insurance type, and surgical procedure. In evaluating complications between patients that underwent ORIF or arthroplasty, Chi-square analysis was conducted to evaluate the potential variation between the surgical groups on the basis of infection, post-hemorrhagic anemia, cardiac complications, respiratory complications, device-related complications, post-operative shock, hematoma formation, pulmonary insufficiency, DVT, any in-hospital complication and mortality.

A logistic regression model controlling for age, gender, Deyo score, and type of procedure was utilized to identify independent predictors of any complication, extended length of stay, and greater hospital charges. Extended length of stay and greater hospital charges were defined as ≥ 60th percentile of the overall cohort. The level of significance was set to *p* value < 0.05. All analysis was performed using IBM SPSS Statistics 24. There was no potential source of bias during this analysis. In order to investigate the association of age and the complications of interest, increased length of stay, total charges, and the occurrence of any complication, an additional analysis was performed on a subset of the dataset containing only patients older than 45 years of age.

## Results

### Overall patient demographics

Analysis of NIS identified an estimated 1413 patients with open proximal humerus fractures with a mean age of 47.62 ± 23.2 years and 59.4% of patients being males. A plurality (28.9%) of patients had private insurance and the majority (50.3%) were white. The most common causes of fractures were falls, gunshots, motor vehicle accidents, and suicide attempts. Detailed demographics are reported in Table 1.

**Table 1:** Demographics of all patients with proximal humerus fractures

Patient demographics	All patients with proximal humerus fractures
Sample size	1413
Mean age (years)	47.6
Deyo index	0.41
Gender	
Male	59.40%
Female	39.90%
Race	
White	50.30%
Black	16.60%
Hispanic	8.80%
Other	3.50%
Data missing	20.80%
Insurance	
Private insurance	28.90%
Medicare	27.40%
Self-pay	16.10%
Medicaid	15.10%
No charge	1.60%
Other	10.30%

The most common procedures were: ORIF (*n* = 662), arthroplasty (*n* = 68) and open reduction without internal fixation (*n* = 33). All available procedures are listed in Table 2.

**Table 2:** Most common procedures for open proximal humerus fractures

	Procedure	*n*
Procedure	ORIF	662
	Arthroplasty	68
	Shoulder hemiarthroplasty	59
	Reverse shoulder arthroplasty	5
	Total shoulder arthroplasty	4
	Open reduction without internal fixation	33
	Internal fixation without reduction	28
	Closed reduction without internal fixation	26
	External fixation	22
	Amputation through humerus	9
	Other/not coded	565
	Total	1413

### ORIF vs arthroplasty

Seven hundred thirty patients who underwent either ORIF (*n* = 662) or arthroplasty (*n* = 68) procedures were further analyzed (Table 3). ORIF patients were younger than arthroplasty patients (48.6 ± 21.8 vs 64.9 ± 17.7, *p* < 0.001) with a lower Deyo score (0.41 ± 0.87 vs 0.7 ± 0.97, *p* = 0.012). In terms of gender, 57.9% of patients undergoing ORIF vs 30.5% of patients undergoing arthroplasty were male (*p* < 0.001). Race varied, with the majority being white in both groups; however, 15.3% of ORIF patients were black vs 0% of arthroplasty (*p* = 0.004). There was a difference in health insurance coverage between the groups with 33.0% of ORIF patients being covered by private insurance vs most arthroplasty patients (55.7%) covered by Medicare (*p* < 0.001) (Table 3).

**Table 3:** Demographic comparisons of patients with proximal humerus fractures that had ORIF or arthroplasty surgical interventions

Patient demographics	Surgical procedure		
	ORIF	Arthroplasty	*p* value
Sample size	662	68	–
Mean age (years)	48.6	64.9	*<* *0.001*
Length of stay (days)	7.86	7.44	0.833
Total charges	$76,998	$64,133	0.360
Deyo index	0.41	0.70	*0.012*
Gender			
Male	57.9%	30.5%	*< 0.001*
Female	42.1%	69.5%	
Race			
White	70.6%	86.4%	*0.004*
Black	15.3%	0%	
Hispanic	10.5%	9.1%	
Other	3.6%	4.5%	
Insurance			
Private insurance	33.0%	19.7%	< *0.001*
Medicare	28.2%	55.7%	
Medicaid	14.2%	1.6%	
Self-pay	14.0%	8.2%	
No charge	1.4%	1.6%	
Other	9.3%	13.1%	

Statistically significant *p* values are in italics

### Surgical complications and hospital course

There was no difference in total complication rate between ORIF and arthroplasty (21.4% vs 18.0%, *p* = 0.535). Specifically, there was no difference with respect to post-hemorrhagic anemia (15.4% vs 13.1%, *p* = 0.636), pulmonary insufficiency (4.2% vs 0%, *p* = 0.102), infection (1.1% vs 1.6%, *p* = 0.677) and device-related complications (0.5% vs 1.6%, *p* = 0.231). The length of stay in ORIF patients was 7.86 ± 14.9 days while length of stay in arthroplasty patients was 7.44 ± 12.1 days (*p* = 0.833). Total charges were comparable, with a mean total cost of $76,998 for ORIF patients and $64,133 for arthroplasty (*p* = 0.833). Despite the fact that one patient from the ORIF group died, the mortality rate was comparable (0.2% vs 0%, *p* = 0.761) (Table 4). When stratified by age, patients greater than 45 years of age showed the same trends.

**Table 4:** Common surgical complications in ORIF and arthroplasty

Complication	ORIF	Arthroplasty	*p* value
Post-hemorrhagic anemia	15.4%	13.1%	0.636
Pulmonary insufficiency	4.2%	0.0%	0.102
Infection	1.1%	1.6%	0.677
Cardiac complications	0.9%	0.0%	0.456
Respiratory complications	0.9%	1.6%	0.575
Device-related complications	0.5%	1.6%	0.231
Hematoma	0.5%	0.0%	0.599
Post-operative shock	0.3%	0.0%	0.668
Digestive complications	0.3%	0.0%	0.668
Urinary complications	0.3%	0.0%	0.668
Deep vein thrombosis	0.2%	0.0%	0.761
Pulmonary embolism	0.0%	0.0%	–
Total complication rate	21.4%	18.0%	0.535

Note, total complication rate does not represent a sum of all complications, but represents rate of any complication following surgery. The row labels do not represent an inclusive sample of all possible surgical complications

Logistic regression revealed that, when using ORIF as a reference, the type of surgery (ORIF vs arthroplasty) was not a predictor of any complications (OR = 0.67 [95% CI 0.33–1.35], *p* = 0.266), extended length of stay (OR = 1.01 [95% CI 0.58–1.78], *p* = 0.967), or high hospital charges (OR = 1.39 [95% CI 0.68–2.86], *p* = 0.366). Age was the only independent predictor of sustaining any complication (OR = 1.01 [95% CI 1.003–1.02], *p* = 0.006). Similarly, increased Deyo score was a predictor of extended length of stay (OR = 1.24 [95% CI 1.01–1.51], *p* < 0.036) and greater hospital charges (OR = 1.31 [95% CI 1.02–1.69], *p* < 0.034). Finally, increased age was a predictor of decreased hospital charges (OR = 0.98 [0.97–0.99], *p* = 0.001) (Table 5). Subsequent logistic regression on patients older than 45 revealed the same trends, except for the association between age and the occurrence of any complication, which was not significant within this subset.

**Table 5:** Output of binary logistic regression models to identify independent predictors of any complications, extended length of stay and greater hospital charges

Outcome	Predictor	OR [CI, 95%]	*p* value
Any complications	Age	1.01 [1.00–1.02]	*0.023*
	Gender	1.04 [0.71–1.53]	0.841
	Deyo index	1.10 [0.90–1.34]	0.335
	ORIF vs arthroplasty	0.67 [0.33–1.35]	0.266
Extended length of stay (> 4 days)	Age	0.99 [0.99–1.01]	0.655
	Gender	0.95 [0.69–1.31]	0.758
	Deyo index	1.24 [1.01–1.51]	*0.036*
	ORIF vs arthroplasty	1.01 [0.58–1.78]	0.967
Hospital charges > $39,387	Age	0.98 [0.97–0.99]	*0.001*
	Gender	0.81 [0.53–1.22]	0.303
	Deyo index	1.31 [1.02–1.69]	*0.034*
	ORIF vs arthroplasty	1.39 [0.68–2.86]	0.366

Statistically significant *p* values are in italics

## Discussion

This study found that for open proximal humerus fractures, the hospital course of patients undergoing either ORIF or arthroplasty is comparable. There was no statistical difference in the inpatient complication rate between the two procedures (21.4% vs 18.0%, respectively). Age played a minimal role in predicting complications or hospital charges. However, increased underlying comorbidities prior to surgery, as reflected by the Deyo score, was the only predictor of increased length of stay and higher hospital charges in this patient population. Patients who underwent ORIF were younger and more likely to be black, male, and insured by a private insurance company when compared to the arthroplasty cohort.

The findings of this study are comparable to those previously reported in the literature on closed fractures. Thorsness et al. [11] reported no differences in 30-day complication rates and length of hospital stay between hemiarthroplasty and ORIF. This study also reported that patients with proximal humeral fractures undergoing ORIF were younger than patients undergoing hemiarthroplasty. This is most likely due to the quality bone stock available in patients under 65 years old [12]. This may also be due to the desire to preserve more bone in individuals who participate in higher risk activities that may lead to future complications or reoperations. As the results of this study concluded, others also found that ORIF patients were more likely to be male [11, 13].

Petrigliano et al. [14] reported that males over 65 years old with preexisting comorbidities had the highest short-term complication rates; this is in line with our findings. However, the mortality rate in our results was comparable between the groups. This is also consistent with Thorsness et al. [11], who found that the mortality rate between the two surgeries was not statistically significant. On the other hand, Neuhaus et al. [15] found a 1.9% increase in hospital deaths in patients who underwent ORIF compared to arthroplasty. Regarding insurance coverage, Zhang et al. [16] showed that arthroplasty patients had an overall higher Medicare rate, which is similar to the results of this study (28.2% for ORIF vs 66.7% for arthroplasty). This difference is most likely due to the increased age and eligibility of patients undergoing arthroplasty.

Although the data reached conclusions similar to those found in previous research, conflicts still remain. Some studies available found that patients who receive shoulder arthroplasties had reduced complications and revision rates when compared to those who received ORIF [17]. In contrast, Cvetanovich et al. [18] reported that ORIF had the fewest 30-day complications of the three procedures. Manoli et al. [19] also found that patients receiving total shoulder arthroplasty (TSA) were more likely to have higher total hospital charges than patients receiving either hemiarthroplasty or ORIF, and that patients receiving TSA had a shorter length of stay. This directly contrasts with our findings that there was no difference between ORIF and arthroplasty with regards to either hospital charges or length of stay.

According to Bell et al. [20], the rate of surgical treatment for proximal humerus fractures is increasing, but it is currently unknown if equivalent results can be anticipated with open fractures. With an increasing elderly population in the future, it is important to investigate the options for this type of open fracture. This study found no difference in inpatient complication rates between arthroplasty and ORIF for open proximal humerus fractures. To our knowledge, this was the first study to compare these two surgical options for the treatment of these types of fractures.

### Limitations

One limitation of this study is that the Nationwide Inpatient Sample (NIS) only contains information from the patient’s hospital stay, and does not contain any follow-up information or functional outcome of these patients. Additionally, due to the nature of the NIS dataset, we were unable to comment on the classification and types of fractures associated with these surgical interventions, as these data points are not collected within the NIS. Further, the database contains little information regarding the technique used for the ORIF and arthroplasty procedures. Nevertheless, our conclusions are drawn from a large number of patients over 15 years. To our knowledge, our study reports on one of the largest sample sizes of patients with open proximal humerus fractures within the literature, and aligns with views and conclusions from the vast majority of pertinent literature.

## Conclusion

The results from this study help clarify the previous unknowns between two possible procedures, ORIF and arthroplasty, used to treat open fractures of the proximal humerus. While ORIF might be desired for bone preservation in young patients, this study confirms that the risk of in-hospital complications is the same as in those who undergo arthroplasty. The number of comorbidities represented by the Deyo score seems to increase the inpatient complication rate and length of stay. Specifically reporting on open fractures also gives this data a unique perspective not discussed in other studies. Ultimately, further analysis needs to be performed, comparing the long-term complications and clinical outcomes, before further recommendations can be made.
